# CSM-Toxin: A Web-Server for Predicting Protein Toxicity

**DOI:** 10.3390/pharmaceutics15020431

**Published:** 2023-01-28

**Authors:** Vladimir Morozov, Carlos H. M. Rodrigues, David B. Ascher

**Affiliations:** 1 School of Chemistry and Molecular Biosciences, University of Queensland, St Lucia, QLD 4072, Australia; 2 Computational Biology and Clinical Informatics, Baker Heart and Diabetes Institute, Melbourne, VIC 3004, Australia

**Keywords:** protein toxicity, sequence, deep-learning

## Abstract

Biologics are one of the most rapidly expanding classes of therapeutics, but can be associated with a range of toxic properties. In small-molecule drug development, early identification of potential toxicity led to a significant reduction in clinical trial failures, however we currently lack robust qualitative rules or predictive tools for peptide- and protein-based biologics. To address this, we have manually curated the largest set of high-quality experimental data on peptide and protein toxicities, and developed CSM-Toxin, a novel in-silico protein toxicity classifier, which relies solely on the protein primary sequence. Our approach encodes the protein sequence information using a deep learning natural languages model to understand “biological” language, where residues are treated as words and protein sequences as sentences. The CSM-Toxin was able to accurately identify peptides and proteins with potential toxicity, achieving an MCC of up to 0.66 across both cross-validation and multiple non-redundant blind tests, outperforming other methods and highlighting the robust and generalisable performance of our model. We strongly believe the CSM-Toxin will serve as a valuable platform to minimise potential toxicity in the biologic development pipeline. Our method is freely available as an easy-to-use webserver.

## 1. Introduction

Peptides and proteins have emerged as powerful therapeutic options; due to their specificity, selectivity and intrinsic functionalities, they have emerged as treatments to previously undruggable diseases. This has seen biologics gain an increasingly larger market share, routinely occupying the majority of the top 10 most profitable drugs. Despite these advantages and remarkable progress, the development of new biologics faces unique challenges, particularly immunogenicity, toxicity, and stability [1]. Besides peptides and proteins, small molecules are also used in therapy. Their advantage is their small molecular weight which allows them to penetrate cells and target specific proteins inside them. On the other hand, peptides and proteins interact with other proteins on the cell surface. While significant effort has been invested into the prediction and optimisation of peptides and proteins, the assessment of toxicity typically relies on expensive and time-consuming in-vivo assays late in the development process. Comparatively, we have seen that the introduction of rules and predictive algorithms to assess small molecule toxicities has helped to significantly reduce clinical trial failures [2,3,4,5].

To address this, a couple of in-silico methods have been developed recently to predict the toxicity of peptide candidates [6,7,8,9,10]. These approaches have generally relied on features derived from the amino acid sequence, including evolutionary information, amino acid composition and multiple sequence alignments. This has shown that computational approaches could provide early estimates of toxicity for thousands of proteins and peptides; however, these approaches have been limited by the use of undersampled data sets, the need to produce additional sequence features before running them, and the use of obsolete technologies.

In this work, we present the largest and the most up-to-date dataset of experimentally measured protein and peptide toxicities. Using this data, we have adapted a deep learning model, ProteinBERT [11], to develop CSM-Toxin, a predictive model of protein toxicity that relies solely on the amino acid sequence with no additional features. This model makes up part of a framework of our other models that use the Cutoff Scanning Matrix (CSM). Although CSM-Toxin does not use CSM, its inclusion in the name emphasizes that it is a part of the framework. We demonstrate that our approach outperforms previous works and can be a valuable resource for the scientific community in better screening and understanding peptide and protein toxicity. CSM-Toxin is freely available via an easy-to-use webserver at https://biosig.lab.uq.edu.au/csm_toxin (accessed on 17 January 2023).

## 2. Materials and Methods

### 2.1. Datasets

**Figure 1:**
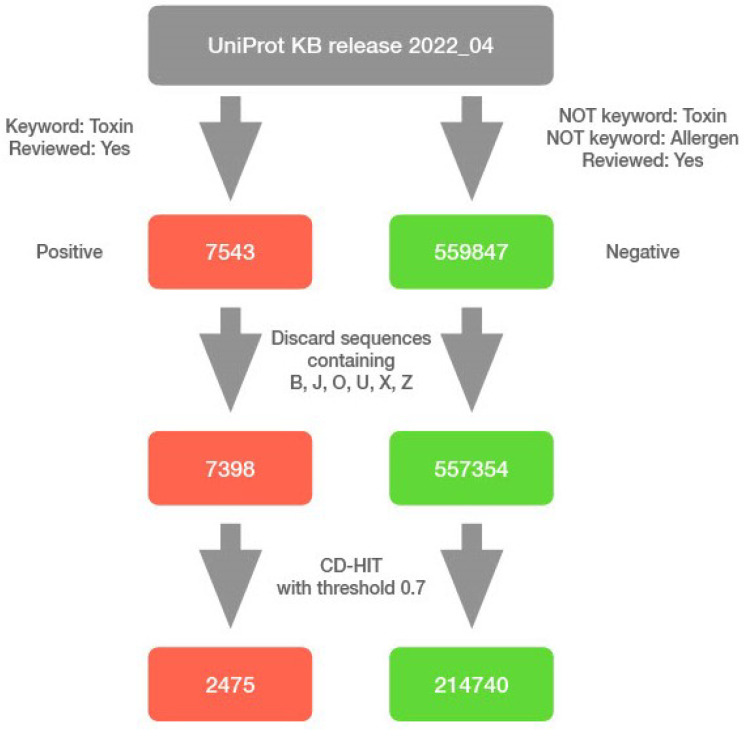
Data processing pipeline. Data were collected from the UniProt KB database (release 2022_04) using a keyword search for toxic and non-toxic proteins. Sequences with non-standard amino acids were discarded and final datasets were clustered for each class separately using CD-HIT.

The data for this study were obtained from UniProt [12] release 2022_04. We identified previously reviewed toxic proteins (positive sample) using the query “(keyword:KW-0800) AND (reviewed:true)” and reviewed non-toxic and non-allergic proteins (negative sample) using query “NOT (keyword:KW-0800) NOT (keyword:KW-0020) AND (reviewed:true)”. The queries resulted in 7543 and 559,847 entries, respectively. We discarded all sequences containing non-standard residue codes, and we used CD-HIT version 4.8.1 [13] with a similarity threshold of 0.7 [14], in order to remove redundant sets of protein sequences. We discarded 143 sequences of longer than 5000 residues, of which one was toxic and the rest were non-toxic, and the longest of them contained over 35,000 residues. Although the model generalises well to work with longer sequences, the training time and memory consumption decreased by several times after discarding these sequences. Nonetheless, the final model can still work with proteins of arbitrary length. Our final curated dataset contains 2475 toxic sequences and 214,740 non-toxic sequences. We used the dataset as is, reflecting inherent biases, with a toxic to non-toxic ratio of approximately 90. The predictive model was solely built using the raw amino acid sequences with no additional features extracted or generated (Figure 1).

We split our curated sequences as follows:

- The 203 toxic and 2337 non-toxic sequences that were uploaded to UniProt after July 2021 and were not present in any datasets used to develop the previous methods. These sequences were used in an independent blind test set for both CSM-Toxin and ToxinPred2, and were not used in training or validation, in order to compare their performance.
- A total of 236 positive and 21,294 negative sequences were taken randomly to form a blind test set. These sequences were not used in training or validation, but only to obtain the final performance metrics.
- The remaining data were equally split in five parts for cross-validation. In each of these splits, 80% of data was used in training, and the remaining 20% was used in validation. The cross-validation performance metrics are averages across these splits.

### 2.2. Model Structure

We used ProteinBERT [11] as the base for our model. ProteinBERT follows the same principle as the natural language processing model BERT (Bidirectional Encoder Representations from Transformers) [15], by treating amino acids as words and protein sequences as sentences. Its main advantage is the use of attention mechanisms, which can capture sophisticated connections between even distant residues.

ProteinBERT was pre-trained using the Masked Language Model technique, where up to 15% of amino acids were hidden (or “masked”, hence the technique name), and the model was expected to recover them based on the context. This technique allows ProteinBERT to capture the connections between amino acids and their surroundings. The pre-training stage was unsupervised, meaning that no labels were provided as inputs to the neural network. The model was expected to extract all the information from the data itself. ProteinBERT was pre-trained on more than 100 million protein sequences derived from the UniProt database.

Although originally inspired by BERT, ProteinBERT has a few differences in its architecture, in particular it has the following two outputs: a binary Global Ontology vector containing 8943 binary values, and Sequence attentions containing 26 values for each input amino acid. In our work, we focused on Global Ontology since what we want is a binary prediction (toxic/non-toxic) based on the whole sequence.

### 2.3. Model Fitting

We fine-tuned the ProteinBERT model by making all the parameters in ProteinBERT non-trainable and trained the whole model for 20 epochs using the Adam optimiser with a learning rate of 0.005 (Figure S1A). After this, ProteinBERT was trainable again, and the whole model was trained for 15 more epochs with a lower learning rate of 0.0001 and weight decay (Figure S2B). Lowering the learning rate and introducing weight decay were necessary steps to prevent the appearance of gradients with large norms. Such gradients would introduce significant changes to model weights and affect patterns learned by the model during pre-training. As our data are imbalanced, we used class weights to inform the model to pay more attention to positive entries.

### 2.4. Cross-Validation and Blind Testing

**Figure 2:**
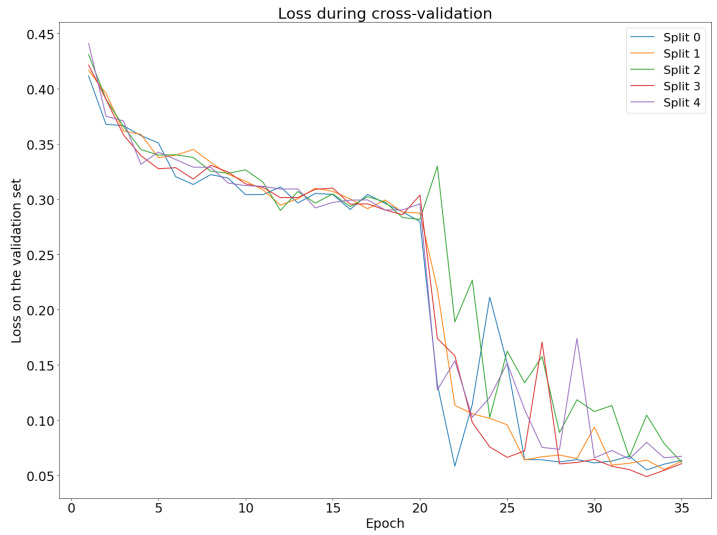
Loss (binary cross entropy) on the validation set during cross-validation. Different colours represent different splits. For the first 20 epochs, ProteinBERT weights were not updated. After it was made trainable, we observed a significant decrease in the loss. Note how at several points the loss tends to increase for 2–4 epochs before decreasing further. This behaviour meant the early stopping technique interrupted the training process.

In order to assess our choices of hyperparameters and model structures, we used 5-fold cross-validation. Model structure and hyperparameters were chosen based on performance on the validation set (Figure 2).

The model outputs a value of between 0 and 1 (the activation function after the last layer is sigmoid). To obtain a binary prediction, a threshold must be chosen, where all values less than this threshold will be treated as negative predictions and all values greater or equal than the threshold will be treated as positive predictions. During cross-validation, we varied thresholds from 0.01 to 1.0 with steps of 0.01 and examined the corresponding changes in MCC (Matthews Correlation Coefficient), AUC (Area Under Curve), and Precision and Recall on the validation sets. The main metric used to choose the final model architecture and hyperparameters is MCC. It has been previously shown to be a more appropriate metric for assessing predictions in unbalanced data, such as the one presented in this study, unlike accuracy, precision and recall [16].

## 3. Results

### 3.1. Data

In this research, we have built the largest dataset of curated toxic and non-toxic sequences. Proteins in our positive data set were derived from 756 different organisms, while proteins in the negative set come from 7091 organisms. The distribution of organisms that provide toxic proteins is shown in Figure S5.

In addition, based on Gene Ontology terms (Figure S5), we observed that over 25% of proteins in the positive set (toxic) area were associated with *toxin activity* functions. However, a range of other biological functions were present, including blocking of ion channels, interaction with blood and cells and blocking receptors.

### 3.2. Model Structure

**Figure 3:**
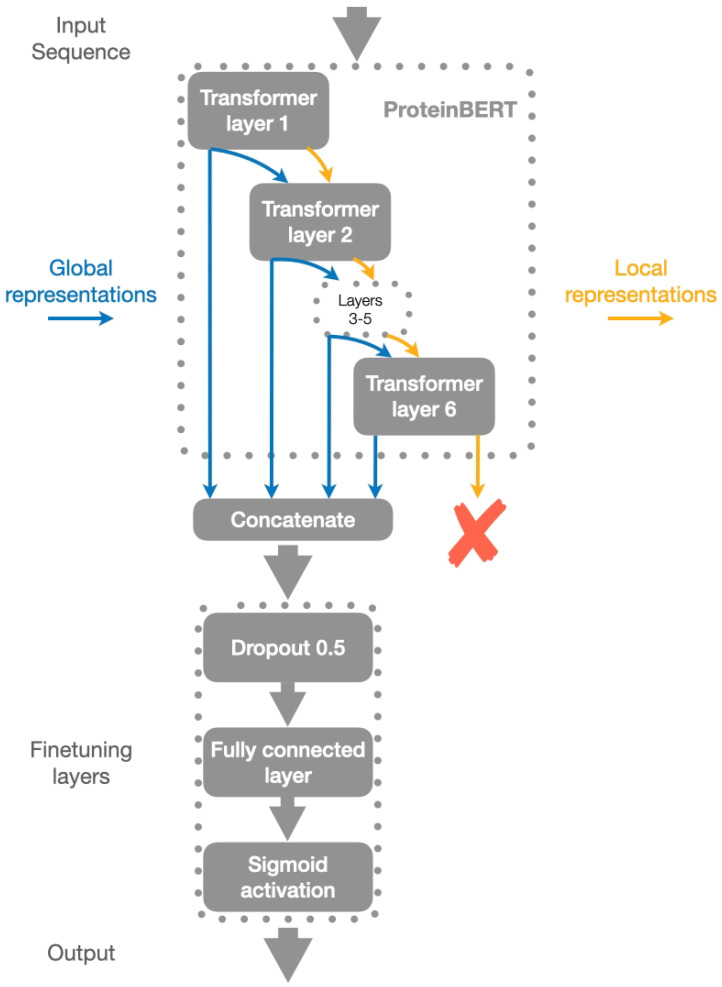
CSM-Toxin model structure. Instead of working with the model output (i.e., the output of the last Transformer layer), we chose to combine the outputs of each of the six Transformer layers. We added a Dropout and a fully connected layer to this combined output. Global representations (or Global Ontology) are marked blue; Local representations are marked orange.

In our work, we took the Global Representation outputs of each of the six Transformer layers and used them for model output. This proved to yield significantly better performance compared to using just the original model output. We added a Dropout layer with a probability of discarding a connection of 0.5 to prevent the model from overfitting. Then, a fully connected layer with Sigmoid activation and a single output was added. This layer’s output was used to determine toxicity: if the output is greater than a given threshold, the protein is classified as toxic, and non-toxic otherwise. The overall structure is shown in Figure 3.

### 3.3. Model Performance

As demonstrated in Figure S2, the maximal value of MCC on cross-validation splits usually corresponds to a threshold of between 0.9 and 1.0.

To find a more precise value for the best threshold, we varied thresholds from 0.9 to 1.0 with steps of 0.001 and examined the corresponding changes in the metrics. As demonstrated in Figure S3, the maximum value of MCC is achieved at a threshold of 0.968.

The final results were obtained by training the model on the entire training data using the same hyperparameters as during cross-validation and validating it on the blind test sets. There are two blind sets: one consists of all the sequences uploaded to UniProt after July 2021, and the second was randomly sampled from the remaining data corpus. The first one has a non-toxic to toxic ratio of 11, while the second one has a ratio of 90.

During five-fold cross-validation, our model achieved an MCC of 0.66 and AUC of 0.86. Evaluation metrics are reported as the mean values for all the five splits. Detailed results are shown in Table S1 and we observe that the model remained consistent across the different splits for all metrics.

On the non-redundant blind test set, our final model achieved an MCC of 0.64 and an AUC of 0.86, consistent with the performance during cross-validation. All metrics, including Precision and Recall, are summarised in Table S2.

### 3.4. Comparison with Other Methods

Our other blind test set was created from the sequences uploaded to UniProt after July 2021, which means that none of the previous models encountered these sequences. We also excluded them from our training and validation sets. Unfortunately, we were unable to run many previous methods on our blind test set. Some of these methods (ToxClassifier [17]) use outdated and no-longer-supported tools like Python 2; some of them (Toxify [18]) were unable to make predictions for all the sequences in the blind set, processing only 1796 sequences out of 2540; others (ToxClassifier [17], ToxDL [8]) have inaccessible servers or we were unable to obtain predictions from them. We considered only models that work with proteins; there are a few more models designed to work with peptides which we did not explore.

A comparison with previous models on this blind set is summarised in Table 1. For our predictive model, we achieved an MCC of 0.67, outperforming ToxinPred2 with an MCC of 0.46. Based on the precision metric, it appears that our model has significantly less false positive predictions than ToxinPred2, perhaps due to the abundance of negative examples in the dataset. Nonetheless, our model predicts slightly more false negative values and thus has a lower recall.

On this blind set, our model misclassified 133 sequences, of which 81 were false positives and 52 were false negatives. These sequences come from 51 and 32 different organisms, respectively. We were unable to find any correlation between the organism and probability of its toxin being misclassified.

### 3.5. Web-Server

The predictive model is freely available as an easy-to-use web-server and Application Programming Interface (API) at https://biosig.lab.uq.edu.au/csm_toxin (accessed on 17 January 2023). The server front end was developed via Materialize CSS framework version 1.0.0, and the back end was built in Python 3.6 via the Flask framework (version 0.12.3). It is hosted on a Linux server running Nginx (version 1.23.0).

On the submission page, users are required to provide protein sequences or upload a file in a FASTA format (Figure S6A). If provided upon submission, an email will be sent to notify the user when their submission is processed.

In the output page (Figure S6B), results are shown as a downloadable table with one entry per protein sequence in the input. For each entry, predictions are shown alongside a set of general physicochemical properties calculated using the Peptides package [19]. In addition, for each entry in the results table, a button is available to help users investigate the output of the attention heads from our model. Higher values indicate regions in which a given attention layer is deemed more related to the toxic activity of the protein.

## 4. Discussion

The ability to rapidly screen for peptides and proteins with potential toxicity has broad implications not only in the biologic development pipeline, but also for genome wide screening to identify novel toxins that may have interesting clinical or biological properties. In this work, we have curated, to our knowledge, the largest data set of toxic and non-toxic proteins. We analysed the performance of prior predictive approaches on newly characterised sequences. This revealed that predictive performances deteriorated significantly from their published results, indicative of overfitting and poor generalisability. Interestingly, we noticed that 2344 sequences in the ToxinPred2-positive main dataset were not present in UniProt positive reviewed sequences, which may have impacted their training results.

To address this gap, we describe CSM-Toxin, a new approach for protein toxicity estimation. Unlike prior approaches, our model relies only on the protein sequence itself and does not require any additional information. CSM-Toxin was able to accurately and robustly identify toxic proteins, outperforming previous approaches across multiple independent blind test sets.

While training our model, we encountered an interesting behaviour of the early stopping technique, which is used to prevent the model from overfitting. If the loss or any other user-specified metric does not improve on the validation set for several epochs, the algorithm stops training and restores the model to the state with the best result. In our case, early stopping did not allow the model to train for more than five epochs and we decided to investigate the impact of turning off early stopping. Interestingly, the loss on the validation set started increasing after five epochs. However, it was not a steady increase; the loss on the validation set fluctuated for about nine epochs, but then suddenly dropped to 0.05 and continued decreasing. It seems that early stopping prevented the model from getting out of a local minimum.

An interesting observation is that after unfreezing ProteinBERT and training the whole model for 15 epochs, MCC does not fluctuate very much, irrespective of which threshold we choose. This could mean that the model is confident in its predictions, where the majority of true negative values receive a score of less than 0.05 and the majority of true positive values receive a score of greater than or equal to 0.95.

To facilitate easy implementation, our model is freely available by API and through an easy to use web interface at https://biosig.lab.uq.edu.au/csm_toxin (accessed on 17 January 2023), and source code is available at https://bitbucket.org/ascherslab/csm-toxin/src/master/data_processing/ (accessed on 17 January 2023). We anticipate that CSM-Toxin will provide a valuable resource for future efforts to characterise peptide and protein toxicity.

## Figures and Tables

**Table 1:** Performance comparison between CSM-Toxin and ToxinPred2 on a non-redundant blind test set. CSM-Toxin outperforms ToxinPred2 for all metrics, except for Recall, showing a more balanced performance.

Metric	CSM-Toxin	ToxinPred2
MCC	**0.67**	0.46
Precision	**0.65**	0.32
Recall	0.75	**0.84**
AUC	**0.86**	0.84

## Data Availability

All the data generated in this research is publicly available at https://bitbucket.org/ascherslab/csm-toxin/src/master/data_processing/ (accessed on 17 January 2023), as well as instructions to use and reproduce it.

## Supplementary Materials

The following are available online at https://www.mdpi.com/article/10.3390/pharmaceutics15020431/s1, Figure S1: Stage 1 (A) and Stage 2 (B) of training the CSM-Toxin architecture; Figure S2: Dependence of MCC, AUC, precision and recall on thresholds from 0.01 to 1.0; Figure S3: Dependence of MCC on thresholds from 0.9 to 1.0; Figure S4: Distribution of organisms from which toxic proteins were taken; Figure S5: Gene Ontologies of the proteins in our positive dataset; Figure S6: CSM-Toxin web-server interface; Table S1: Values of metrics obtained during cross-validation; Table S2. Values of metrics obtained during random blind testing.
