# Supplementary material for: CSM-Toxin: A Web-Server for Predicting Protein Toxicity
Source: Pharmaceutics. 2023 Jan 28;15(2):431. doi: 10.3390/pharmaceutics15020431 (PMC9966851; doi:10.3390/pharmaceutics15020431)
Supplement: Supplementary file 1 [file pharmaceutics-15-00431-s001.zip › pharmaceutics-2100259-supplementary.pdf]

Supplementary Materials: CSM-Toxin: A Web-Server for Predicting Protein Toxicity

Vladimir Morozov [1,2], Carlos H. M. Rodrigues [1,2] and David B. Ascher [1,2] *

Figures

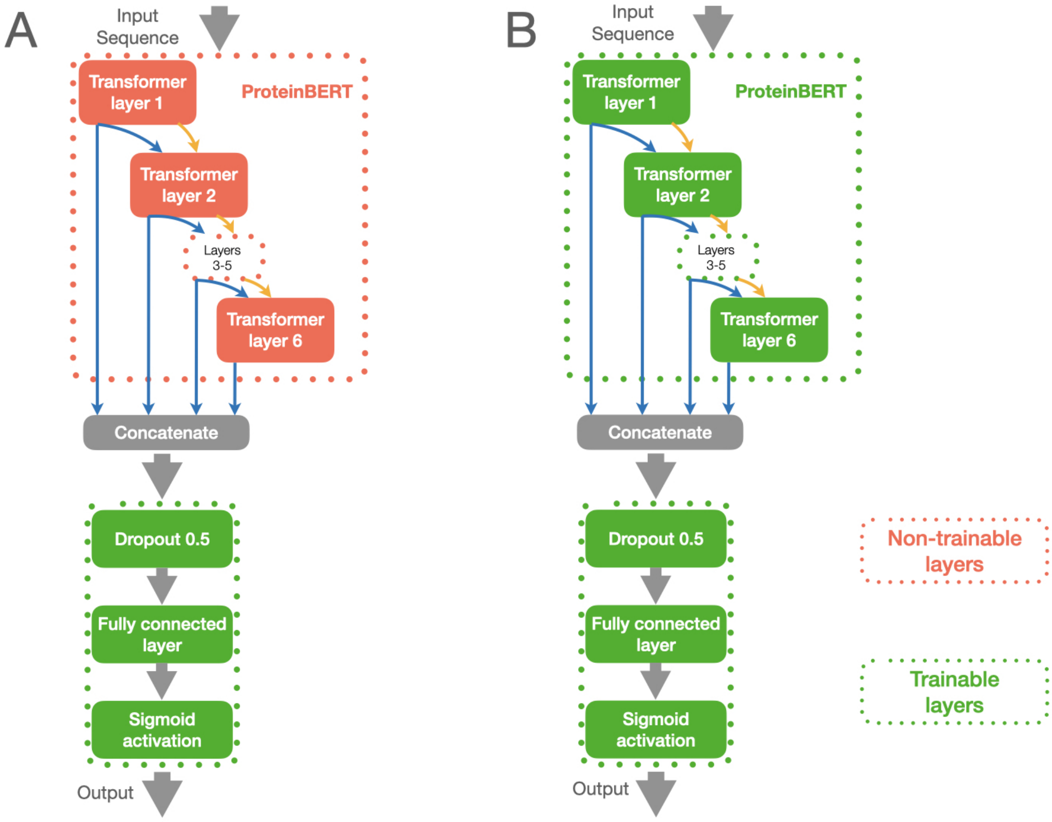

Figure S1. Stage 1 (A) and Stage 2 (B) of training the CSM-Toxin architecture. During the Stage A, only the fully-connected layer is being trained. All the ProteinBERT layers are made non-trainable. During the Stage B, all the ProteinBERT layers are made trainable. The whole model is being trained with a lower learning rate.

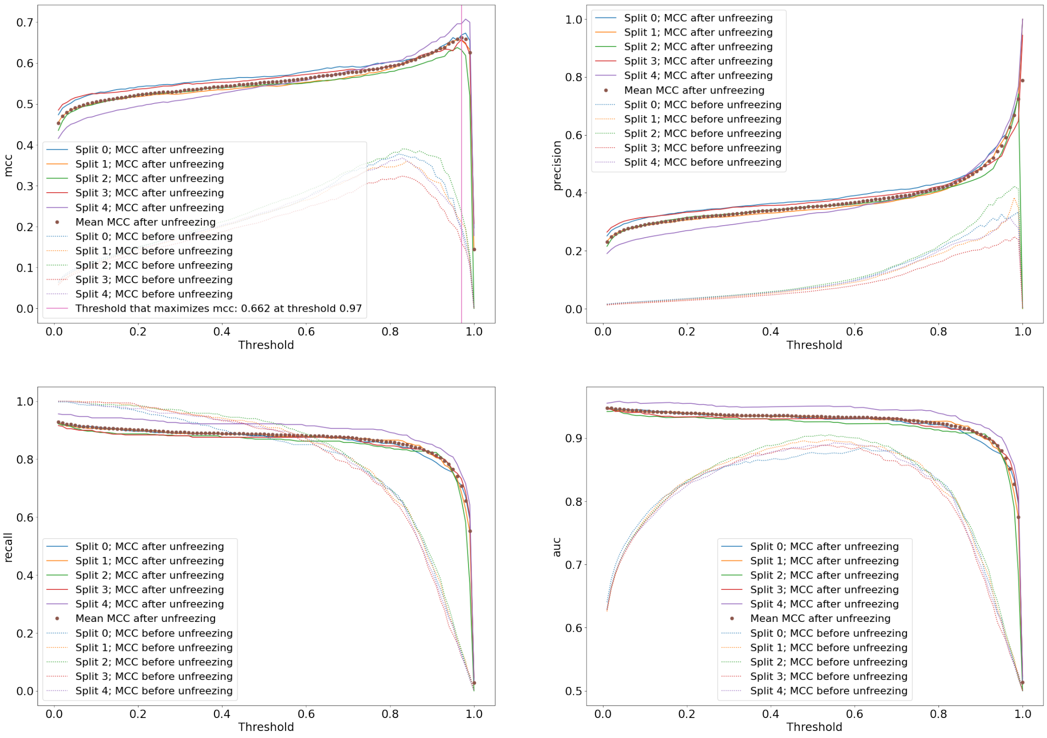

Figure S2. Dependence of MCC, AUC, precision and recall on thresholds from 0.01 to 1.0. Different colours represent different splits. Solid lines represent the metrics after unfreezing and training ProteinBERT, while dotted lines represent MCC after training only the finetuting layers.

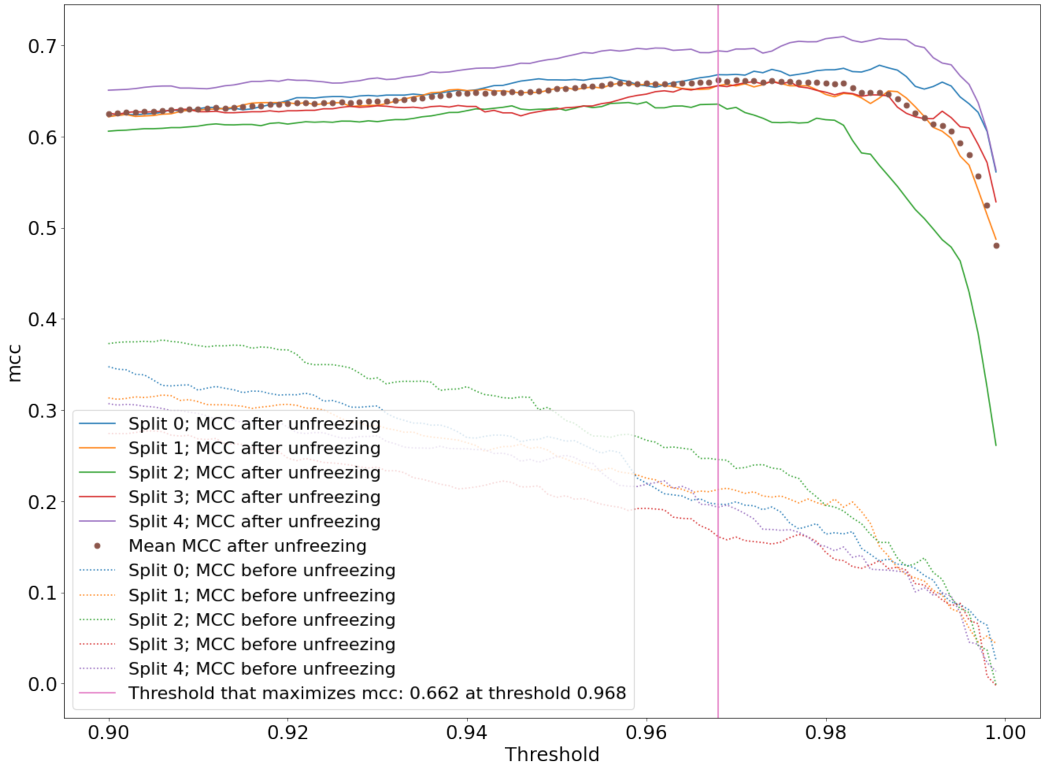

Figure S3. Dependence of MCC on thresholds from 0.9 to 1.0. Different colours represent different splits. Solid lines represent the metrics after unfreezing and training ProteinBERT, while dotted lines represent MCC after training only the finetuting layers.

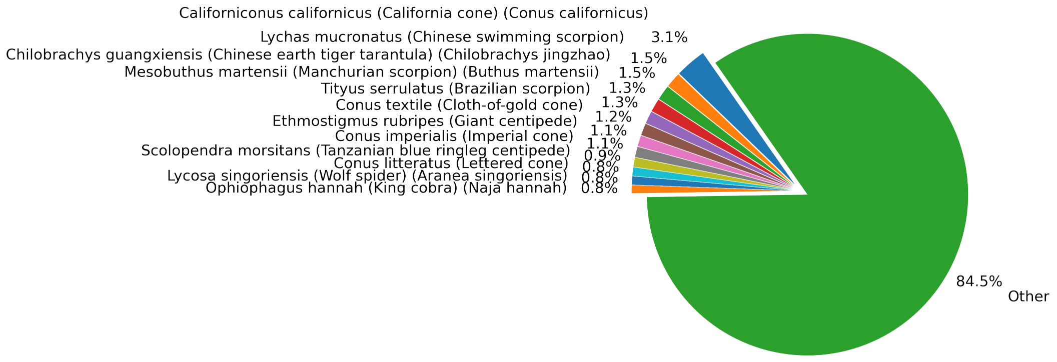

Figure S4. Distribution of organisms from which toxic proteins were taken.

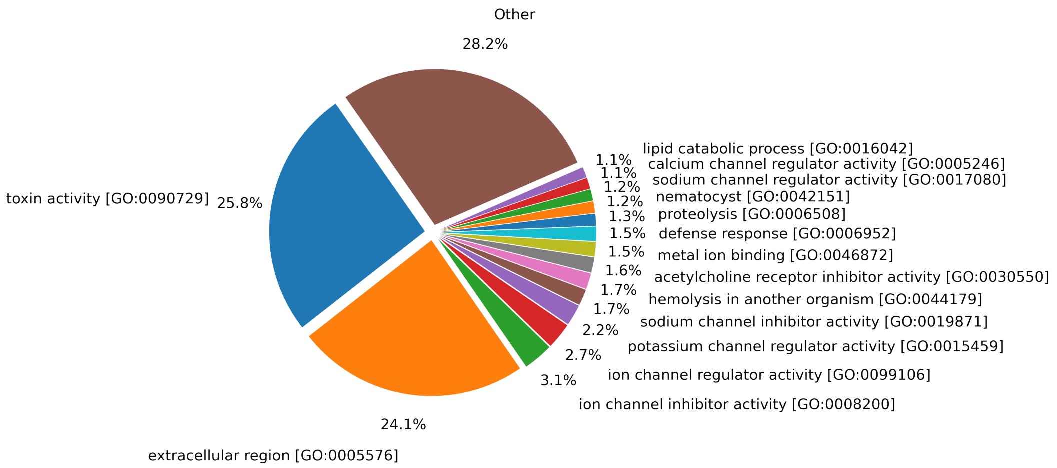

Figure S5. Gene Ontologies of the proteins in our positive dataset.

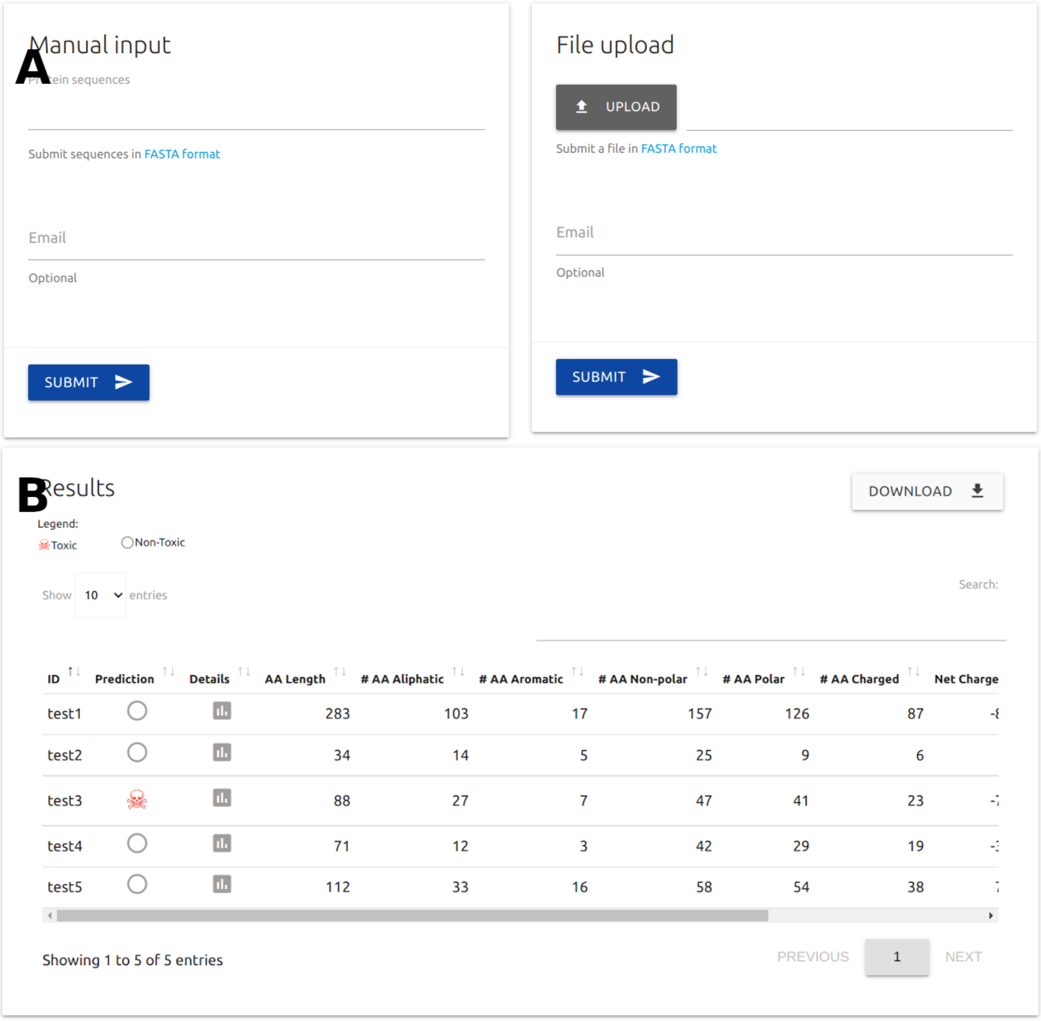

Figure S6. CSM-Toxin web-server interface. A) shows the input page of the server where users are required to provide protein sequences using the text input or upload a file in FASTA format. If provided upon submission, an email will be sent to notify the user when the job finishes processing. B) depicts the results page. Results are shown as a downloadable table with one entry per protein sequence followed by the predictions and additional generic physicochemical properties.

Tables

Metric	Split 1	Split 2	Split 3	Split 4	Split 5	Mean
MCC	0.67	0.66	0.64	0.66	0.69	0.66
Precision	0.63	0.62	0.61	0.59	0.64	0.62
Recall	0.72	0.70	0.67	0.74	0.76	0.72
AUC	0.86	0.85	0.83	0.87	0.88	0.86

Table S1. Values of metrics obtained during cross-validation.

Metric	CSM-Toxin
MCC	0.64
Precision	0.56
Recall	0.73
AUC	0.86

Table S2. Values of metrics obtained during random blind testing.
